# Ionic Liquid Directed Mesoporous Carbon Nanoflakes as an Effiencient Electrode material

**DOI:** 10.1038/srep18236

**Published:** 2015-12-10

**Authors:** Lirong Kong, Wei Chen

**Affiliations:** 1 i-Lab, Suzhou Institute of Nano-Tech and Nano-Bionics, Chinese Academy of Sciences, Suzhou, 215123 P. R. China

## Abstract

Supercapacitors are considered to be the most promising approach to meet the pressing requirements for energy storage devices. The electrode materials for supercapacitors have close relationship with their electrochemical properties and thus become the key point to improve their energy storage efficiency. Herein, by using poly (vinylidene fluoride-co-hexafluoropropylene) and ionic liquid as the dual templates, polyacrylonitrile as the carbon precursor, a flake-like carbon material was prepared by a direct carbonization method. In this method, poly (vinylidene fluoride-co-hexafluoropropylene) worked as the separator for the formation of isolated carbon flakes while aggregated ionic liquid worked as the pore template. The obtained carbon flakes exhibited a specific capacitance of 170 F/g at 0.1 A/g, a high energy density of 12.2 Wh/kg and a high power density of 5 kW/kg at the current of 10 A/g. It also maintained a high capacitance retention capability with almost no declination after 500 charge-discharge cycles. The ionic liquid directed method developed here also provided a new idea for the preparation of hierarchically porous carbon nanomaterials.

Recently, a great attention has been focused on the application of meso and microporous carbons as electrode materials for supercapacitors due to their remarkable properties including easy processability, environmental friendness, and high chemical stability in acidic or basic solutions as well as high performance ability in a wide range of temperatures^1^,^2^. The porous carbon nano electrode materials were usually formed by alkali activation treatment. In order to better manipulate the porous structure of the derived carbon materials and alleviate the environmental pollution, hard or soft templates were used to guide the formed pore structure of as-prepared carbon electrode material, including SiO_2_^3^,^4^, CaCO_3_^5^, NH_4_Cl^6^, polystrene (PS) and many other polymer surfactants (P123, F127 *et al.*)^7^,^8^,^9^,^10^,^11^. Among all kinds of template materials, polymer templates were mostly used since no post-treatment was needed to remove the template after the calcination process. That’s because during the calcination process, the polymer template could quickly and dramatically shrink, and thus the pores were *in-situ* formed. Sun *et al.* reported the preparation of ordered mesoporous carbons via soft-template method. By using different carbon precursors, the pore size was tunable in the range of 3.6-6.2 nm^12^. According to Zhang *et al.*, F127 templated mesoporous carbon structure could reach a high BET surface area of 2660 m^2^/g and a total pore volume of 2.01 cm^3^/g after CO_2_ activation^13^. However, as the prepared mesoporous carbon materials always exhibited a bulk mesoporous phase, the quick immigration of ions into the mesopores was hindered. As a result, when they were used as the electrode materials for supercapacitors, their capacitance were relatively low, especially under high charge-discharge current. In order to resolve this problem, many groups tried to create both macro and meso pores in the carbon matrix or shape them into nanostructures, such as nanofibers or nanotubes to accelerate the immigration process of the electrolyte ions into the mesopores^14^,^15^,^16^,^17^,^18^,^19^. For example, Vinu *et al.* reported the preparation of highly ordered macro-meso porous carbon nitride film by using P123-PS spheres as dual templates^14^. Kalra’s group and Yu’s group prepared mesoporous carbon nanofibers by an electrospinning process before calcination^15^,^16^. Considering the extra experimental steps for the preparation of PS templates or electrospinning the raw material into nanofibers, these methods were a little troublesome. As a result, facile methods for the synthesis of nanostructural mesoporous carbon electrode materials still needed to be investigated.

Recently, many groups focused on ionic-liquid-assisted synthesis of mesoporous inorganic materials since it had good solubility characteristics, strong interaction with inorganic matrix and easy removing process. The porous inorganic nanostrctures templated by ionic liquid (IL) were usually fabricated by electrodeposition, the sol–gel method and the solvothermal route^20^,^21^. The obtained product could keep a highly porous structure after the removing of the IL template. However, up to now, only a few results were reported for the preparation of IL templated porous carbon materials through direct calcination treatment^22^,^23^,^24^.

Herein, we proposed the direct carbonization route to prepare mesoporous carbon nanoflakes by using polyacrylonitrile (PAN) as the carbon precursor, and using poly(vinylidene fluoride-co-hexafluoropropylene) (PVDF-HFP) and ionic liquid (IL) as dual templates. In this method, PVDF-HFP worked as the separator for the formation of isolated carbon flakes while IL worked as the pore template. Moreover, their electrochemical capacitive properties, including specific capacitance, rate performance and cycling stability, were also studied. The method provided here offered a new way for the preparation of hierarchically porous carbon nanomaterials.

## Experimental Process

### Materials

PAN (Mw = 150, 000) and PVDF-HFP (Mw = 400, 000) were obtained from Sigma-Aldrich. All other chemicals were obtained from Sinopharm Chemical Reagent Co., Ltd, which were analytical grade and used without further purification, including N-methylpyrrolidone (NMP), N,N-dimethylfomamide (DMF) and 1-ethyl-3-methylimidazolium tetrafluoroborate (EMIBF_4_). The deionized water was purified through Ultrapure Milli-Q system.

### Preparation of Carbon Nanoflakes

5 g PAN, 0.5 g PVDF-HFP and 1 g EMIBF_4_ were dissolved in 20 mL DMF by stirring for more than 6 h. By casting 2 mL of the above solution on a 7.5 × 2.5 cm^2^ area glass substrate and evaporated the solvent on a heating plate at 80 °C for 2 h, the composite membrane was obtained. In order to obtain the final arrayed carbon nanoflakes, the above composite membrane was calcined at 280 °C for 1 h and then at 700 °C for 1 h under N_2_ atomosphere at a flow of 100 cm^3^ min^−1^ with a 5 °C min^−1^ heating rate.

### Electrochemical Measurement

In a three-electrode system, the test electrode was prepared by loading slurry consisting of 80 wt % active material, 10 wt % carbon black, and 10 wt % poly-(vinylidene fluoride) (PVDF) (in NMP) on a nickel foam and dried at 80 °C for 1.5 h under vacuum. As-formed electrodes were then pressed at a pressure of 10 MPa and further dried in a vacuum oven at 80 °C overnight. The loading mass of active materials on each current collector was 3.0-4.0 mg, and area was 1.0 cm^2^. In the three-electrode system, the sample was used as the test electrode, platinum foil as the counter electrode, Ag/Ag^+^ electrode as reference electrode, and 6.0 M KOH aqueous solution as electrolyte. Cyclic voltammetry curves were obtained in the potential range of -1.0–0 V *vs* Ag/Ag^+^ electrode. Galvanostatic charge-discharge measurements were carried out at 0.1-10.0 A g^−1^ over a voltage range of −1.0–0 V *vs* Ag/Ag^+^ electrode. The specific capacitance of the supercapacitor cell was calculated from the equation of *C*_*cell*_ = *IΔt/m*Δ*V*, where *I* is the discharge current, Δ*t* is the discharge time, *m* is the total mass of active materials in the electrode, and Δ*V* is the voltage drop upon discharge. The energy density (*E*) and power density (*P*) of a supercapacitor cell in the Ragone plots were calculated following the equations of E = 1/2*C*_*cell*_Δ*V*^2^ and P = *E*/Δ*t*, respectively.

### Chracterizations

Transmission electron microscopy (TEM) and Field emission scanning electron microscopy (FESEM) images were recorded by FEI Tecnai G2 F20 S-Twin 200 KV and Hitach S-4800 equipped with an Energy Dispersive Spectrometer respectively. Raman spectra were measured on Horiba JY Labrain HR800 Raman spectroscopy (exciting source: 632 nm laser for Raman in air at room temperature). N_2_ adsorption/desorption analyses were carried out at 77 K using Micromeritics ASAP 2050. Electrochemical performance analyses (cyclic voltammeter (CV) curves and galvanostatic charge-discharge test were recorded by CHI660C electrochemical work station. Thermogravimetric analysis (TGA) was conducted using asystem under dry air with a heating rate of 10 °C min^−1^.

## Results and Disccussions

### Morphology and Formation Mechanism

**Figure 1:**
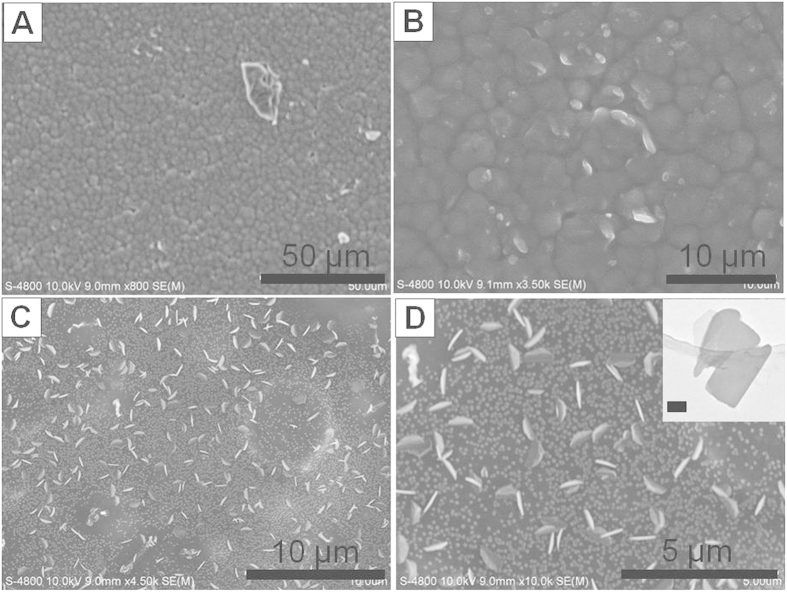
SEM image of as-prepared PVDF-HFP/PAN/IL films: (**A**) under low magnification and (**B**) under high magnification; SEM images of carbon flakes: (**C**) under low magnification and (**D**) under high magnification. TEM image of carbon flakes (inserted in D, scale bar: 250 nm). Experimental conditions for carbon flakes: [PVDF-HFP] = [PAN] = 25 mg/mL, [IL] = 50 mg/mL.

**Figure 2:**
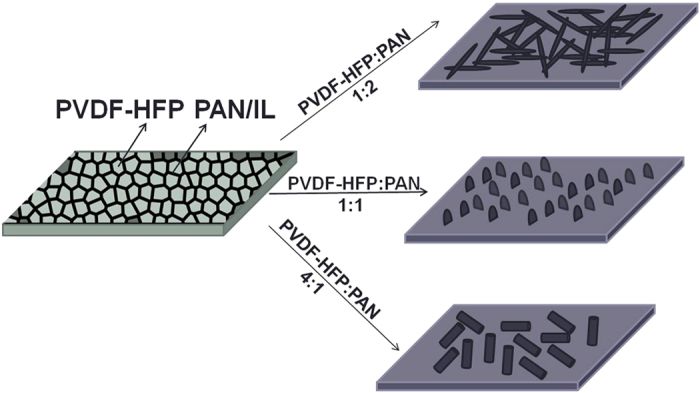
Formation mechanism of carbon nanostructures.

The top view SEM images of as-prepared and calcined PVDF-HFP/PAN/IL films were shown in Fig. 1. As shown in Fig. 1A, the as-prepared PVDF-HFP/PAN/IL composite film exhibited a chapped morphology, which is composed of isolated “islands” and the gaps between them. After calcination, the isolated islands disappeared and some standed carbon flakes which existed in similar arrangement way with the gaps were left (Fig. 1). Moreover, among the carbon flakes, some small carbon dots were formed on the surface of lower-layer carbon substrate. According to these results, we proposed the suggested mechanism for the formation of these carbon flakes and in the manuscript below, we would prove our suggested mechanism by more experimental data. The suggested formation mechanism was as below: since PVDF-HFP and PAN were of different miscibility with IL, the composite film was separated into two phases after being dried at 80 °C. One phase is PVDF-HFP islands which have poor miscibility with IL and the other one is PAN/IL gaps as PAN has good miscibility with IL. The good miscibility originated from the hydrogen bond interaction between EMI^+^ of IL and the –CN group of PAN. After calcination, PVDF-HFP greatly shrinked into small carbon dots while PAN/IL was carbonized into mesoporous carbon flakes (Fig. 2). As PAN shrinked less than PVDF-HFP upon calcination, the carbon flakes were a lot larger than the carbon dots. Since IL could be removed under high temperature, the IL existed in PAN leaded to the formation of the mesopores in the obtained carbon flakes (Figure S1). By tunning the weight ratio of PVDF-HFP to PAN, the nanostructure could be well tuned from nanofibers to nanoflakes, and finally to nanorod-like products. Among them, since the carbon flakes exhibited the best electrochemical energy storage performance, we mainly concentrated our work on its formation mechanism and electrochemical property investigation.

**Figure 3:**
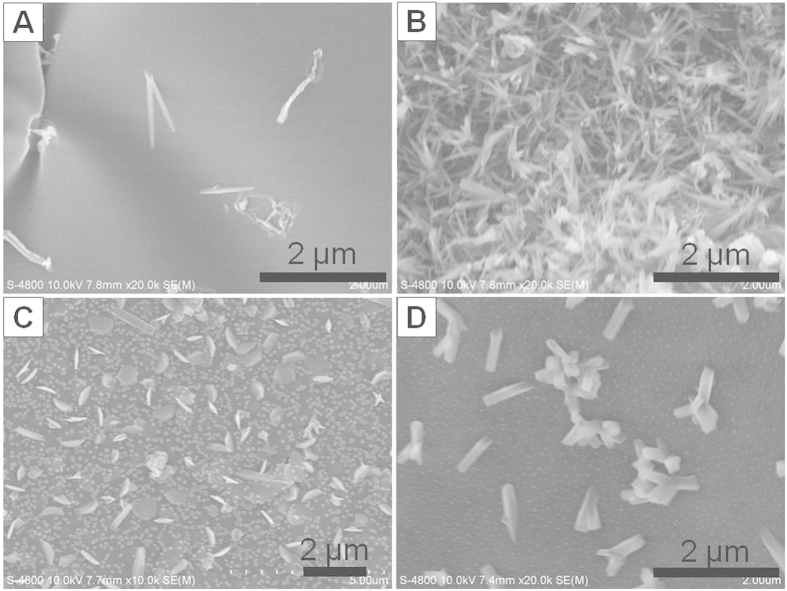
SEM images of carbonized PVDF-HFP/PAN/IL films with different ratios of PVDF-HFP to PAN: (**A**) 1:4; (**B**) 1:2; (**C**) 2:1; (**D**) 4:1. Other conditions: [PVDF-HFP+PAN] = 50 mg/mL; [IL] = 50 mg/mL.

In order to prove our suggested mechanism for the formation of carbon flakes, firstly, we compared the SEM morphologies of the carbon flakes with carbonized PVDF-HFP/PAN, PAN, PVDF-HFP, PAN/IL and PVDF-HFP/IL films. From supplementary Figure S2, it could be clearly observed that no flake-like morphology appeared in the final products and only some small pores or irregular particles formed on their surface due to the evaporation of IL and shrinkage of the polymer. Secondly, we tuned the used amount of IL in the composite films to investigate their influence on the morphology of the final products. As shown in supplementary Figure S3, along with the increase of the concentration of IL in film-casting solution, the carbon flakes became larger and larger, taller and taller, and finally formed irregular short rod-like product when the concentration of IL reached to 100 mg/mL. This was because IL worked as a template agent for the phase separation. In detail, when no IL was added in the PVDF-HFP/PAN solution, the two polymers were well mixed into a homogenous solution and the phase separation did not happen in the obtained composite film. Along with the increase of the added amount of IL, the phase separation became more and more obvious, and the PAN/IL gaps became deeper and larger. As a result, the final carbon flakes became larger and taller. When the concentration of IL reached to 100 mg/mL, the formed structure of PVDF-HFP/PAN/IL composite film was totally changed and the rod-like nanostructures instead of carbon flakes were observed in the final product. Moreover, in order to investigate the roles of PVDF-HFP and PAN played in the formation process of carbon flakes, the SEM images of the carbonized PVDF-HFP/PAN/IL films with different ratios of PAN to PVDF-HFP were given out in Fig. 3. As shown in Fig. 3, the carbon flakes could only be obtained when the ratios of PAN to PVDF-HFP were 1:1 and 1:2. At other ratio values, the product exhibited nanofibrous morphologies. This indicated that only when the concentrations of PAN and PVDF-HFP were close, the composite film could exhibit the chapped morphology for the formation of carbon flakes. When anyone of them was overdose, the templated structure for the formation of carbon flakes would be destroyed. As a result, after calcination, only fibrous morphology could be obtained.

**Figure 4:**
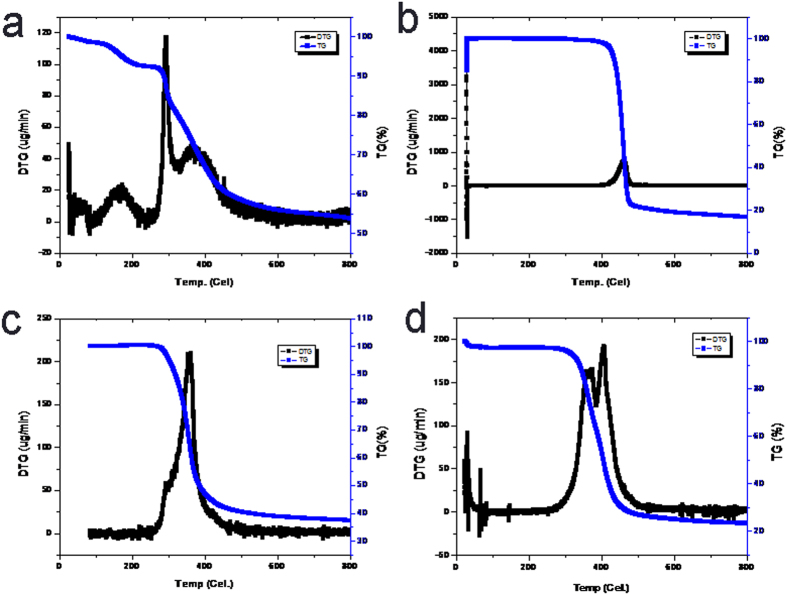
DTA and TGA curves of (**a**) PAN; (**b**) PVDF-HFP; (**c**) PAN/IL and (**d**) PVDF-HFP/IL films.

In order to further confirm the suggested mechanism, different shrinkage extents of PVDF-HFP and PAN upon calcination were proved by their DTA and TGA curves. As shown in Fig. 4, the weight retention of PAN after calcination was about 53% while that of PVDF-HFP was about 17% (right axis). Moreover, the weight retention of PAN/IL and PVDF-HFP/IL were 38% and 22% respectively. These results further confirmed the different shrinkage extents after calcination, which contributed to the formation of carbon flakes. Since the densities of calcined PVDF-HFP and PAN/IL were similar, high weight retention corresponded to a large volume. As a result, the carbon flakes were a lot larger than the carbon dots produced from PVDF-HFP.

**Figure 5:**
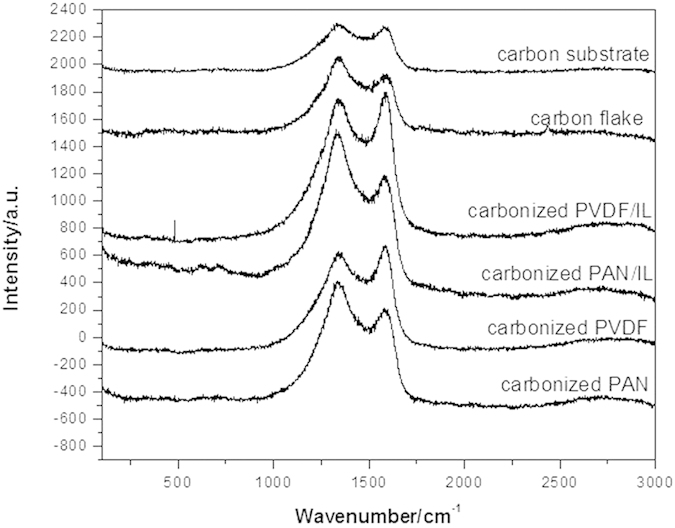
Raman spectra of carbon substrate, carbon flake, carbonized PVDF-HFP/IL, carbonized PAN/IL; carbonized PVDF-HFP and carbonized PAN films.

As described in the part of formation mechanism, the carbon flakes were formed from PAN/IL gaps. As a result, it should exhibit similar Raman spectra with calcined PAN. On the other hand, the carbon substrate under the carbon flakes was mainly composed of calcined PVDF-HFP. From the Raman spectra tested on the carbon flake side and on the carbon substrate side (Fig. 5), it could be clearly observed that the results were in well accordance with our assumption. All of the calcined products exhibited two characteristic peaks of carbon materials, which were the well-documented D band at 1328 cm^−1^ and G band at 1587 cm^−1^, corresponding to the disordered graphitic carbon and the E2g vibration of the sp^2^ bonded carbon atoms^25^. The PVDF-HFP based carbon materials, including calcined PVDF-HFP and PVDF-HFP/IL exhibited a obviously higher D band and a relatively lower G band, indicating a low graphitization degree while the PAN based carbon materials, including calcined PAN and PAN/IL, exhibited a slightly higher G band than D band, which indicated a much higher graphitization degree than PAN based carbon materials. Since the Raman spectrum tested from the carbon flake side exhibited very similar intensity distribution of D band and G band to calcined PAN and PAN/IL, it is believed that the carbon flakes were mainly derived from PAN. Similarly, as the carbon substrate side exhibited analogous intensity distribution of D band and G band to PVDF-HFP based carbon materials, it could be inferred that the carbon substrate was mainly derived from PVDF-HFP. These results also proved our suggestion about the formation mechanism of the carbon flakes.

**Figure 6:**
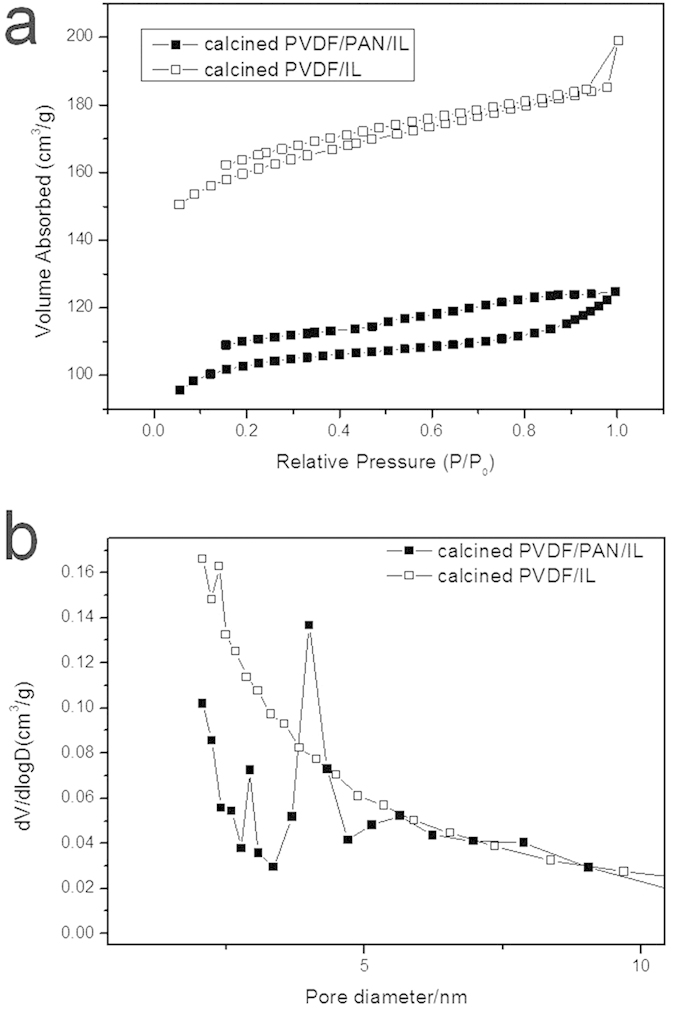
(**a**) Nitrogen adsorption–desorption isotherm and (**b**) pore-size distribution of calcined PVDF-HFP/PAN/IL and PVDF-HFP/IL.

The surface area and pore diameter distribution of the as-prepared product was obtained by N_2_ adsorption/desorption analysis. Since the surface area of calcined PAN/IL was too small to be analyzed by Micromeritics ASAP 2050, only the analyzed results of calcined PVDF-HFP/PAN/IL and PVDF-HFP/IL were presented in the manuscript. As shown in Fig. 6, both the isotherms of carbon flakes and calcined PVDF-HFP/IL exhibited unclosed loops which were due to the irreversible adsorption of nitrogen during the analyzing process. According to the BDDT classification, both of the two samples exhibited type IV isotherms with H4 hysteresis loop at a relative high pressure which were due to the incomplete desorption of N_2_ from narrow slit-like pores^26^. The hysteresis loop at middle pressure for carbon flakes indicated the presence of mesopores, which could not be seen in the isotherm of calcined PVDF-HFP/IL. This point was more obvious in the pore diameter distribution spectra shown in Fig. 6b. In detail, the calcined PVDF-HFP/IL exhibited only one peak centered at 2.37 nm, while the carbon flakes exhibited two high peaks centered at 2.93 and 4.00 nm respectively. The hierarchically porous structure of the calcined PVDF-HFP/PAN/IL originated to its unique mesoporous flake-like nano morphology. The calculated BET surface areas of calcined PVDF-HFP/PAN/IL and PVDF-HFP/IL were 317.01 m^2^/g and 494.47 m^2^/g. Although the latter sample exhibited a higher surface area, its monodispersed pore structure hindered the immigration of the electrolyte ions during the charge-discharge process and thus its specific capacitance was much lower than the carbon flakes.

### Electrochemical Properties

**Figure 7:**
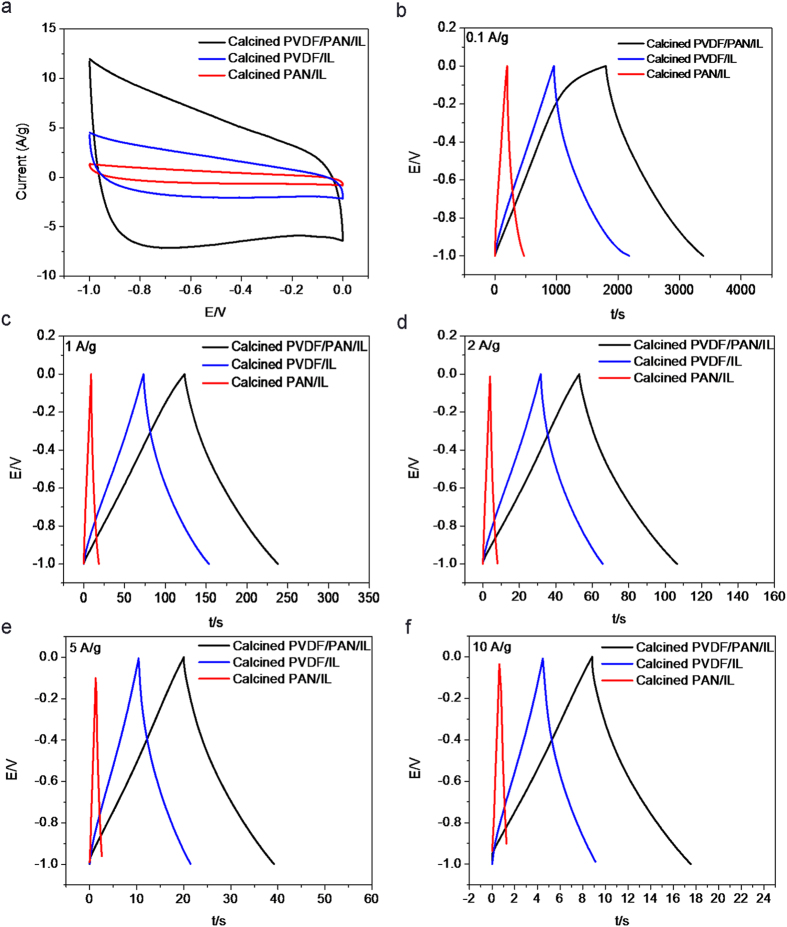
Electrochemical performance of the supercapacitors based on calcined PVDF-HFP/PAN/IL, calcined PVDF-HFP/IL, and calcined PAN/IL electrode materials. (**a**) CV curves at a scan rate of 0.1 V s^−1^ in the potential range from −1 to 0 V; (**b–f**) Galvanostatic charge-discharge curves at different current densities.

The electrochemical capacitive properties of the mesoporous carbon flakes were evaluated by means of CV, galvanostatic charge-discharge and cycling-life tests in three-electrode systems, and were compared with those of calcined PAN/IL and PVDF-HFP/IL. As shown in Fig. 7a, the CVs of all the electrode materials exhibited nearly rectangular shape in the potential range of -1 ~ 0 V, demonstrating their electrical double layer capacitive properties. Among them, the CV curve of mesoporous carbon flake based electrode exhibited larger enclosed area than the other two, indicating its better capacitive performance. Specific capacitances of the three calculated according to the galvanostatic charge-discharge curves (Fig. 7b–f) and the calculated results at different charge-discharge current densities were given in Fig. 7a. The galvanostatic charge-discharge tests were also performed in a potential range from −1 to 0 V. The almost symmetrical charge and discharge curves demonstrated their good and stable capacitive behavior in the wide potential window. As shown in Fig. 7a, at the current density of 0.1 A/g, the carbon flake based electrode exhibited a specific capacitance of 170 F/g, which was much higher than calcined PVDE/IL and PAN/IL, which were 109.1 and 23.6 F/g respectively. Along with the increase of the charge-discharge current density, the specific capacitance of the electrode materials decreased due to the decreased charge-discharge time which was not enough for the immigration of all the electrolyte ions into the electrode. However, the specific capacitance of carbon flake based electrode was still kept a lot higher than the other two kinds of electrode materials. Their different capacitive properties were mainly determined by their different nanostructures. The mesoporous carbon flake exhibited exposed and hierarchically porous morphology which facilitated the immigration of the electrolyte ions into the pores of the electrode material, and thus the electrical double-layer capacitance was improved. Although the calcined PVDF-HFP/IL exhibited larger surface area and pore volume than mesoporous carbon flake, its monodispersed pore structure in the bulk carbon phase hindered the quick immigration of the electrolyte ions, and thus its electrical double-layer capacitance was much lower than that of the carbon flake. For calcined PAN/IL, its low surface area s well as its bulk phase morphology leaded to its low specific capacitance.

To understand the role of nitrogen functionalities in capacitive performance, the atom percentage of nitrogen in the obtained products calcined under different temperatures (600, 700 and 800 °C) were characterized by Energy Dispersive Spectrum. The results indicated that the atom percentages of nitrogen were 7.68%, 4.04% and almost undetectable in the products calcined under 600, 700 and 800 °C. High nitrogen content usually corresponded to high pseudo capacitance^16^. However, in our products, their capacitive performances were not in good correspondence with their nitrogen contents. The specific capacitances were 136.6, 170 and 123.3 F/g for the products obtained under 600, 700 and 800 °C (Figure S4). This may be due to the increased conductivity of the obtained products under high calcination temperature. Considering the effects of both conductivity and nitrogen content, carbon flakes calcined under 700 °C exhibited the highest specific capacitance.

**Figure 8:**
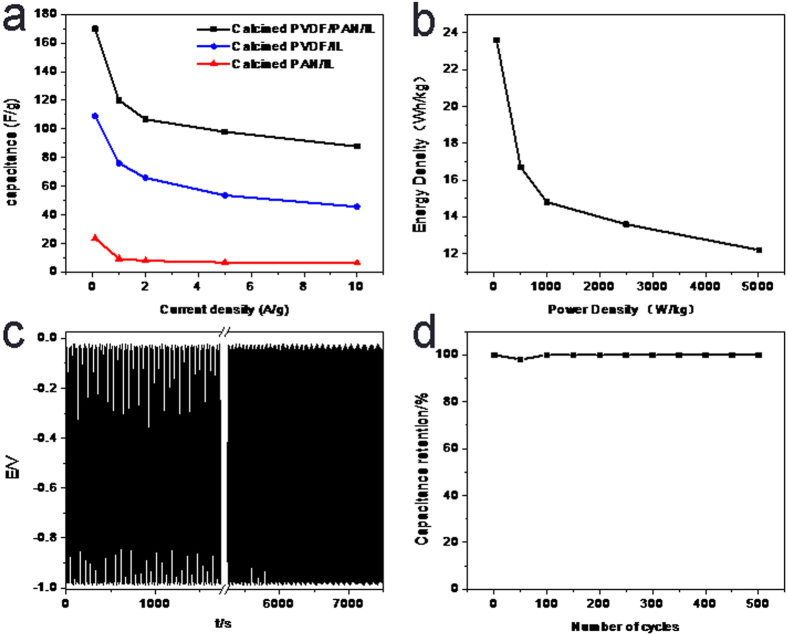
(**a**) Specific capacitance at different current densities; (**b**) Ragone plot of supercapacitors based on RGO/PANI/MWCNT/IL electrode films; (**c,d**) capacitance retention over 500 cycles at 10 A g^−1^.

Ragone plots of the carbon flake electrode based supercapacitor was shown in Fig. 8b, which exhibited a high energy density of 23.6 Wh/kg at the current density of 0.1 A/g, with the power density of 50.2 W/kg. Along with the increase of the charge-discharge current density, the energy density decreased and the power density increased. Owing to the facile ion transportation process for the carbon flake electrode based supercapacitor, the energy density was still high under high charge-discharge current density, which was 12.2 Wh/kg at the current density of 10 A/g with the power density of 5 kW/kg. Moreover, the endurance galvanostatic charge-discharge experiment was also carried out to study the cyclic stability of the carbon flake based electrode. As shown in Fig. 8c,d, the capacitance exhibited almost no declination after 500 cycles.

## Conclusions

In summary, a typical kind of mesoporous flake-like carbon materials was proposed by a direct carbonization method, in which the IL and PVDF-HFP were used as the surfactant-like agent and PAN was used as the carbon precursor. By using this method, the final product could exhibit a hierarchically porous structure which facilitated the ion transportation during the charge-discharge process and thus afforded them high electrochemical capacitive properties. Electrochemical studies demonstrated their specific capacitance of 170 F/g, as well as high energy density and power density, which were 12.2 Wh/kg and 5 kW/kg at a current density of 10 A/g. The carbon flake electrode also showed excellent cyclic stability, as its specific capacitance exhibited almost no declination after 500 charge-discharge cycles. The method proposed here may hold promise for preparing more kinds of carbon materials with hierarchically porous structures for low-cost and high-performance energy storage devices.

## Additional Information

**How to cite this article**: Kong, L. and Chen, W. Ionic Liquid Directed Mesoporous Carbon Nanoflakes as an Effiencient Electrode material. *Sci. Rep.* **5**, 18236; doi: 10.1038/srep18236 (2015).

## Supplementary Material

Supplementary Information
