# Supplementary material for: Ionic Liquid Directed Mesoporous Carbon Nanoflakes as an Effiencient Electrode material
Source: Sci Rep. 2015 Dec 10;5:18236. doi: 10.1038/srep18236 (PMC4674753; doi:10.1038/srep18236)
Supplement: Supplementary Information [file srep18236-s1.doc]

**Ionic Liquid Directed Mesoporous Carbon Nanoflakes as an Effiencient Electrode material**

Lirong Kong, Wei Chen﹡

﹡i-Lab, Suzhou Institute of Nano-Tech and Nano-Bionics, Chinese Academy of Sciences, Suzhou, 215123 (P. R. China). E-mail: [wchen2006@sinano.ac.cn](mailto:wchen2006@sinano.ac.cn)

**Supplementary Information**

**
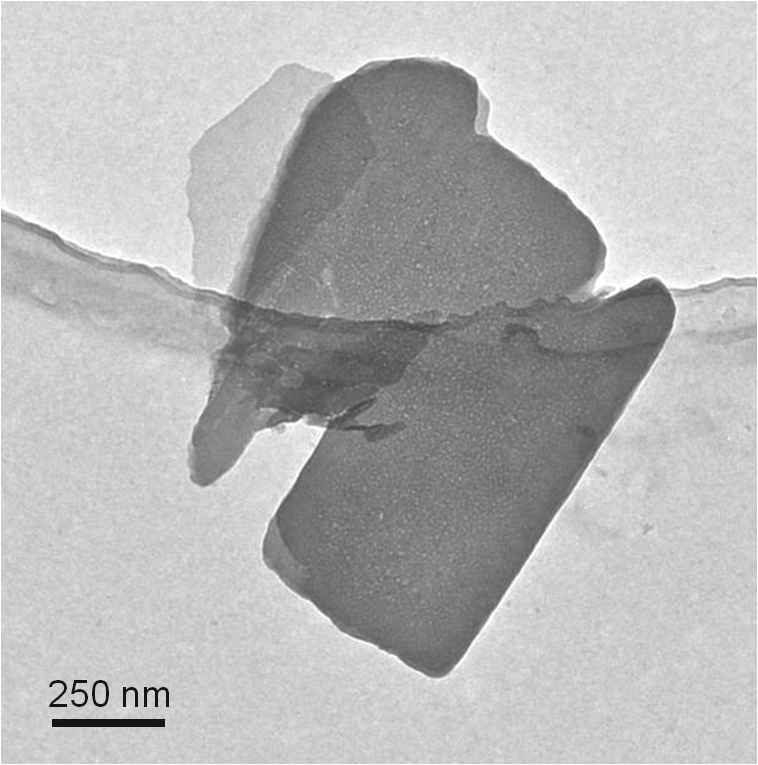
**

**Figure S1.** Magnified TEM image of carbon flakes.


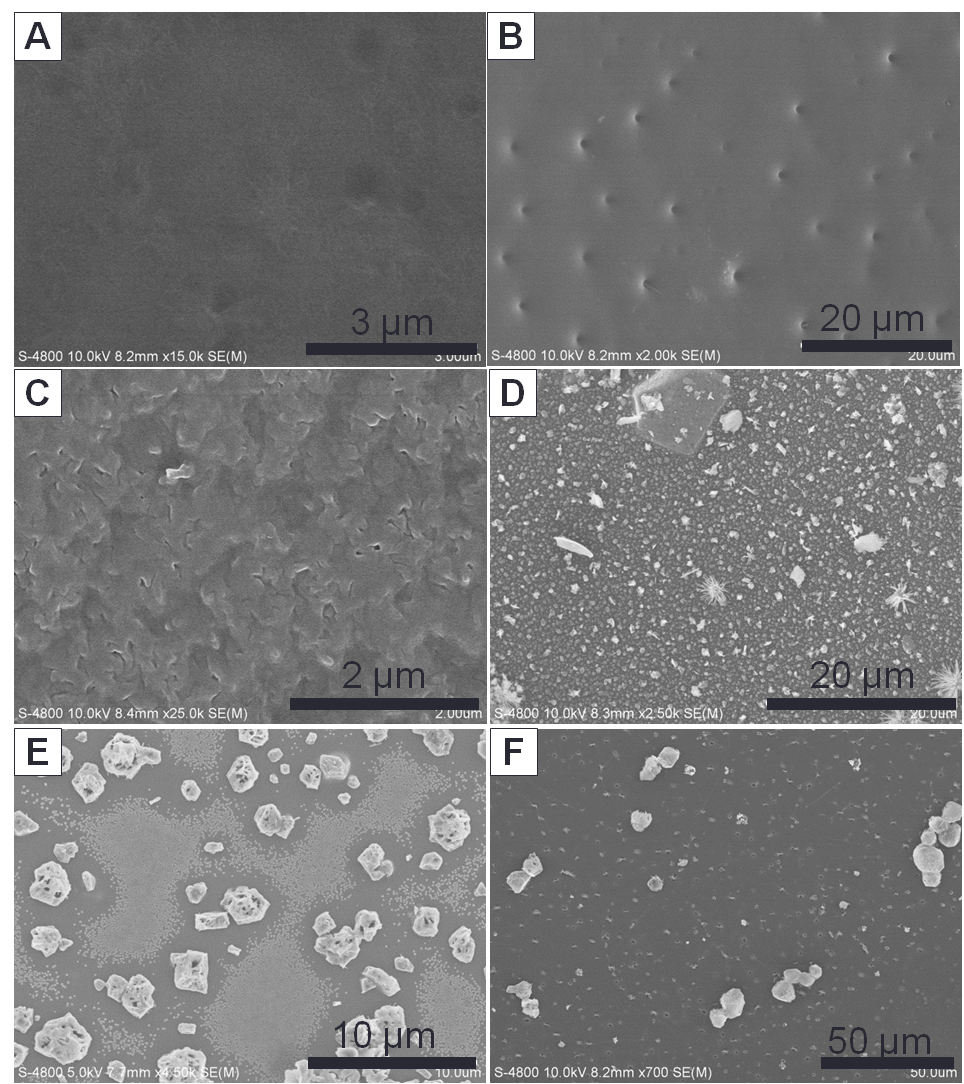


**Figure S2.** SEM images of A) PVDF-HFP/PAN composite film; B) carbonized PVDF-HFP/PAN composite film; C) carbonized PAN film; D) carbonized PVDF-HFP film; E) carbonized PAN/IL composite film; F) carbonized PVDF-HFP/IL composite film.


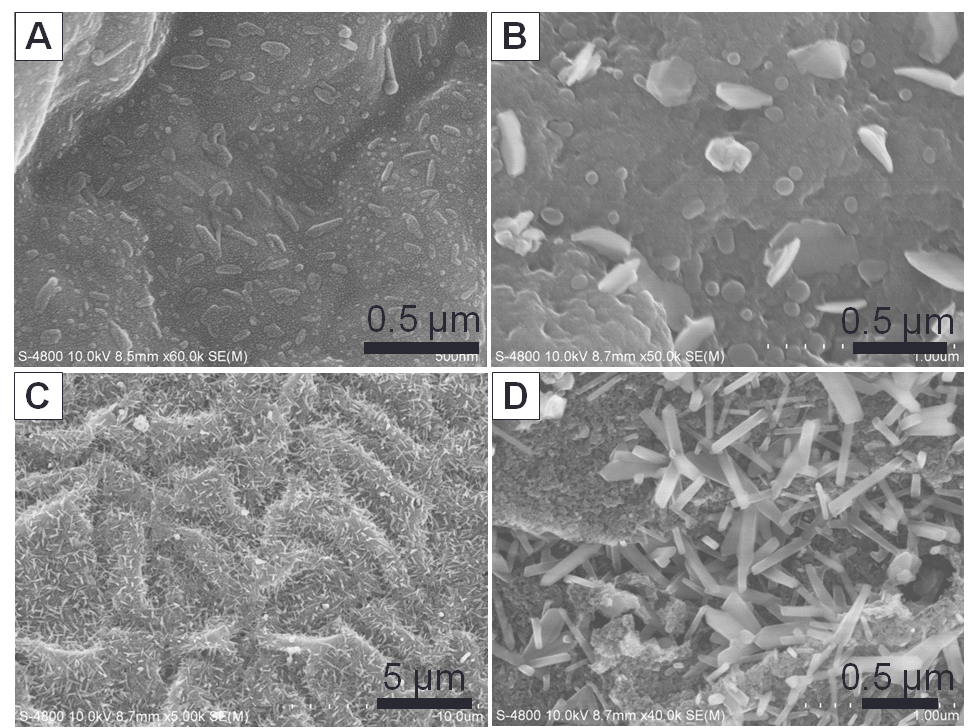


**Figure S3.** SEM images of carbonized PVDF-HFP/PAN/IL films with different concentration of IL in film-casting solution: A) 10 mg/mL; B) 25 mg/mL; C) 100 mg/mL (under low magnification); D) 100 mg/mL (under high magnification). Other conditions: [PVDF-HFP] = 25 mg/mL; [PAN] = 25 mg/mL.


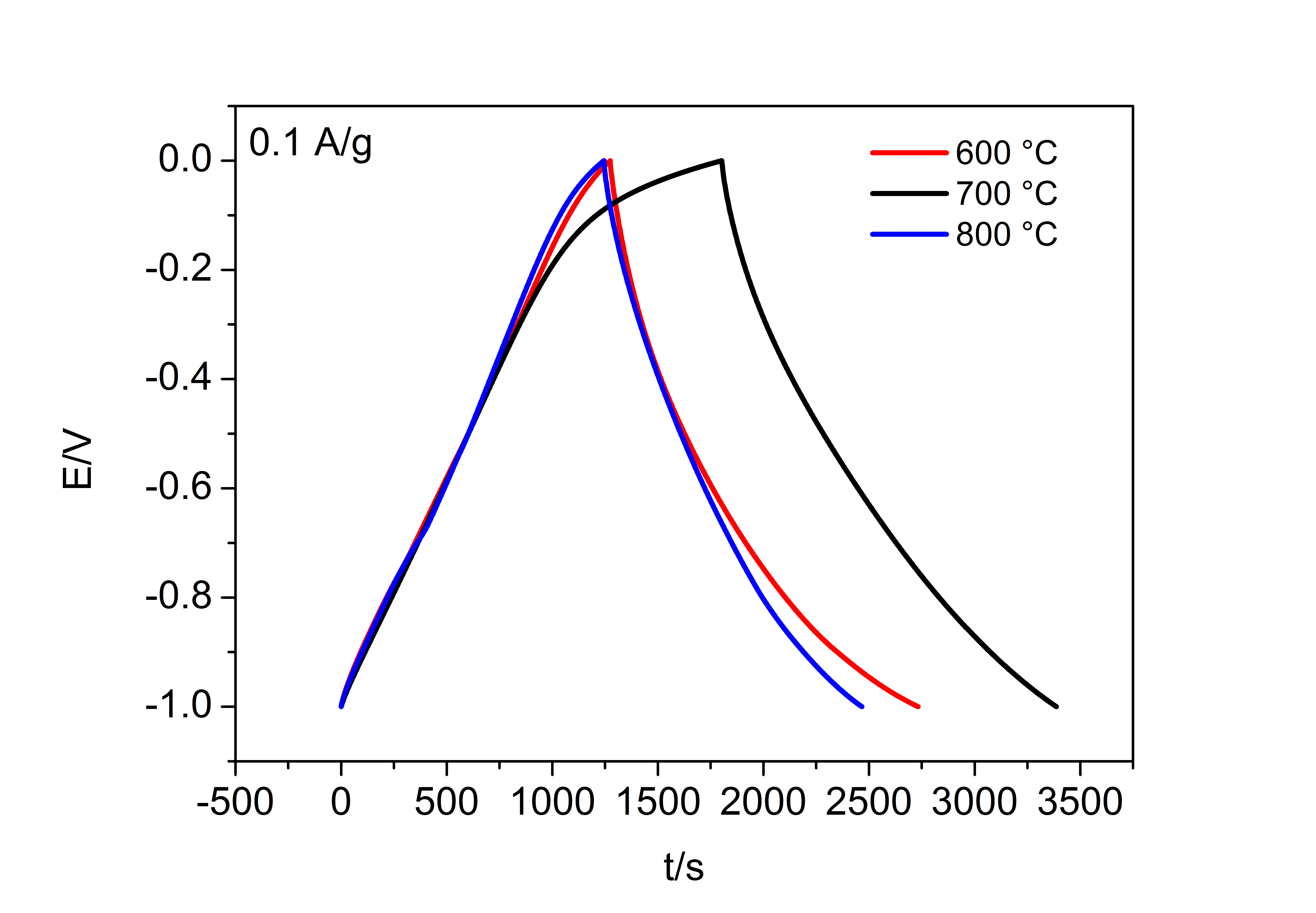


**Figure S4.** Electrochemical performance of the supercapacitors based on calcined PVDF-HFP/PAN/IL calcined under different temperatures.
